# Institutional Pressure and Green Product Success: The Role of Green Transformational Leadership, Green Innovation, and Green Brand Image

**DOI:** 10.3389/fpsyg.2021.704855

**Published:** 2021-10-04

**Authors:** Jian Zhou, Lucinda Sawyer, Adnan Safi

**Affiliations:** ^1^School of Business, Qingdao University, Qingdao, China; ^2^School of Economics, Qingdao University, Qingdao, China

**Keywords:** green brand image, green process innovation, institutional pressure, green transformational leadership, green product success, green product innovation

## Abstract

Rapid economic growth has led to economic activities which have caused extensive environmental damage to the planet. Companies have sought to adapt their business methods to reduce their carbon footprint in order to meet regulations, satisfy consumer preferences and keep up with changing societal expectations. The relationship between institutional pressure and green product performance will be an important issue in corporate green management. This article looked through the lens of green innovation and explored the moderating role of green brand image between green product innovation and new green product success. Utilising the data of 243 managers in Mainland China, structural equation modelling results found that institutional pressure is positively correlated to green transformational leadership, green transformational leadership is positively correlated to green process innovation, green process innovation is positively correlated to green product innovation, green product innovation is positively correlated with new green product success, green brand image moderates the relationship between green product innovation and new green product performance. The research results provide theoretical and practical implications for enterprises to relieve institutional pressure and build specific green competitive advantages.

## Introduction

Rapid economic growth has led to economic activities which have caused extensive environmental damage to the planet (Wang et al., 2021; Xu et al., 2021). This in turn has led to various governmental economic policies and environmental regulations to restrict and regulate activities that cause adverse environmental impacts (Du et al., 2021), this includes carbon emission regulations and taxes. A growing consciousness by consumers of economic activity has also affected consumption choices. Consumer movements calling for greater government and business action in tackling climate change has led to shifts in consumer preferences. Internal and international pressures have also increased pressure on governments and companies to adapt economic practices, in order to reduce or neutralise environmental impact of activities (Xu et al., 2021). This has in turn filtered down into management practices (Weng et al., 2015). Companies have sought to adapt their business methods to reduce their carbon footprint in order to meet regulations, satisfy consumer preferences (Wang et al., 2021) and keep up with changing societal expectations. Firms seek legitimacy within their institutional environment (Zhang et al., 2019), which is influenced by stakeholders, including government actors, competitors, and consumers. In recent years, governments have pushed policies on green growth, including “China’s 13th Five Year Plan,” the “Paris Agreement” (Zhang et al., 2019) and the “Green New Deal” (Schuelke-Leech, 2021). Therefore, firms have had to adapt their practices to accommodate these policies (Qi et al., 2021).

Leaders are the visionary actors in a company and are influential in deciding the direction of the company. There are many factors influencing leader’s decisions as to what direction to take the company in. As governmental and societal pressure increases, and competitors also increase their green activities, leaders will feel ever increasing pressure to conform to the prevailing expectations, and to adapt company processes to conform to the increasing green standards (Zhang et al., 2021). Leaders are an important resource for a company, influential in employee conduct and motivation.

Firms must be able to translate green initiatives into profitable outcomes, in order for policies to translate into sustainable management practices and for the firms to survive. Abiding by government regulations is the minimum a company must do. Companies face societal pressure to implement green activities that go beyond simply government regulations as discussed above (Tu and Wu, 2021). Green development can be expensive. Therefore, a key question is whether firms can achieve growth whilst carrying out environmental or green activities (Zhang et al., 2019). New green product development success is important in moving toward further future sustainable activities (Wang et al., 2021). If green products are not successful in terms of their profitability, there is no economic motivation pushing companies to continue to undertake innovation activities to develop new green products (Huang and Wu, 2010).

Consumers will not necessarily know how green a particular product is from its exterior appearance. Companies that have undertaken extensive efforts to let customers know how green their products are will be able to persuade those customers that their product is superior to competitors because it is seen as sustainable or more environmentally friendly (Xie et al., 2019), even though competitors may have undertaken the same steps to create green products. Therefore a green brand image can create a competitive advantage (Zameer et al., 2020). When consumers choose to buy products there are a multitude of factors involved in a purchase decision. However, if customers do not believe that a company or product is green, it will not associate the new product with being green, nor believe that it is greener than competitor products. A brand can be viewed as a promise to a customer about benefits they will receive from the companies’ product (Lin et al., 2021).

This paper seeks to discover an explanation as to why some companies perform better than others, even if all companies are performing green improvements. This provides useful insight to companies who want to ensure that they achieve a return on green investment and green innovations. Green conscious consumers who compare products will use various cues to aid them in their purchase decision (Liu K.-N. et al., 2020; Chen et al., 2021). For companies who are known for being green, when their products are released onto the market, consumers will believe they are green when compared with other companies that do not have a favourable green image. High levels of trust is associated with green brand image. Customers who view the brand favourably will be more likely to trust the company and its products (Lee and Chen, 2019). Therefore, without any other prior knowledge of the product, consumers will use the brand image to help them evaluate their purchase decision (Vilasanti da Luz et al., 2020). This paper will examine how this relationship works in the context of green product innovations and how successful the new green products are. This will help bridge the knowledge gap for firms wanting to improve the success of new products and justify their green innovation efforts to shareholders.

The question this paper seeks to answer is the following questions: when the level of green brand image in a company is higher, does this also mean the success of new green products will be higher? In other words, does green brand image moderate the relationship between green product innovation and new green product success. This question was tested using structural equation modelling, in which the path coefficients were assessed, β, T-statistic and P-value were reported, and accepted or rejected based on the values.

The paper builds up the theory by assessing past literature to build evidence for attempting to test these new hypotheses. It then uses relevant past literature to build up hypothesis and create a research model. The paper then discuses variable measurements and how data for these measurements is to be collected, namely through the use of a questionnaire. Results are analysed for fit and then hypotheses tested using the abovementioned structural equation modelling. Finally the results are discussed in terms of their contribution to management theory and how they can apply to managers in a practical sense.

In terms of its main contribution, this paper explores how institutional pressure can affect the success of new green products on the market, through the lens of green innovation and green brand image. The main problem this paper will address is the question of how a firm green brand image can affect success of its future products. This will be done through the moderation analysis of green brand image. In other words, the paper will explore to what extent a favourable green brand image can help to increase sales of new green products. This will provide theoretical contribution to the growing body of green literature that is only growing importance as governments and society pushes further in aims of a neutral, or a reversal of negative, environmental impact, to save the planet from irreversible degradation. The paper also provides evidence of how a favourable green brand image can mean more profitable products. Profitable products, as opposed to products that do not create profit, are what keeps a company alive as non-profit-generating companies will not survive in the market.

## Litereture Review

### Institutional Pressure

Institutional pressure can be understood from the angle of Institutional Theory, which states that firms seek legitimacy in their institutional environment. This pressure impacts on a firm’s behaviour (Chen et al., 2018). Many authors note that institutional pressure is a driving factor in adoption of environmental initiatives (Dubey et al., 2015). Institutional pressure comprises of three types, namely, coercive pressure, normative pressure, and mimetic pressure. Coercive pressure includes pressures from governmental actors. Normative pressures refer to pressure from customers and organisations that are non-government related. Mimetic pressure is pressure that results from competitors (Chen et al., 2018). The result of these pressures is what are known as coercive, normative and mimetic isomorphism (Dubey et al., 2015).

Along with dividing the concept into different types, the concept can be explored by looking at the role of institutional pressure as a motivation for green product innovation (GPI). Green product innovation refers to goods or service that have undergone an improvement and has resulted in the product or service having a lower environmental impact than its previous versions (Ilg, 2019). Dangelico (Dangelico, 2016) reviewed the current literature and analysed the main antecedents of GPI. Internal motivations include competitive advantage; cost reduction; market opportunities, such as the increase in market share, opportunities, and new markets; reputation; improved product quality; value and culture considerations; internal stakeholder pressures; green capabilities; risk-aversion and marketing orientation. External factors include regulations and policies; market factors such as pressures from external stakeholders; political and cultural environment, media attention toward firms; networking activity such as industrial initiatives, and cooperation with environmentally conscious partners.

As well as analysing institutional pressure as a motivation behind GPI, institutional pressure can also be explored from the angle of its moderating effect. Dubey et al. (2015) found that institutional pressures have a moderative effect between supplier relationship management and environmental performance and between Total Quality Management and environmental performance at the significant level. Chen et al. (2018) found that institutional pressure in terms of coercive and normative pressure are related positively with green correlation.

### Green Transformational Leadership

Green transformational leadership refers to behaviour in which a leader inspires the green motivation of employees and provides vision and inspiration to allow these employees to meet the company’s green goals (Singh et al., 2020). Numerous authors have explored the effect of leadership on green activity, including green creativity (Li et al., 2020; Zhang et al., 2020), environmental performance (Singh et al., 2020) and strategy (Huang et al., 2021). Much research has concentrated on green transformational leadership through the lens of human resource management by exploring green creativity (Chen and Chang, 2013; Jia et al., 2018; Li et al., 2020). Although some authors have linked green human resources to green innovation, more research is needed linking green transformational leadership and green innovation.

Linking green transformational leadership with a green performance indicator, Chen and Chang (2013) conducted research on green transformational leadership and green product development performance, which is the development performance of products with lower environmental impact, defined by factors such as how the products developed compare to competitors and whether the green development meets its environmental goals. It was found that green transformational leadership has a positive influence on green product development performance. However, the research did not look into how the performance is affected through developments of products, i.e., it did not explore how innovations of green products or processes play a role in leading to greater performance. What was missing was exploring the link between the role of leaders and green performance through the role of innovation that takes place to create new products and achieve a favourable green performance. Similarly, other authors also looked at leadership and performance. Dubey et al. (2015) also looked at the effect leadership on environmental performance, through the lens of operational practices and institutional pressures.

Conversely, some authors have explored the effect of leadership on various green factors to do with green employees, such as creativity and procurement. For example Li et al. (2020) found green transformational leadership affects green creativity through green intrinsic and extrinsic motivation. Liu J. et al. (2020) explored the influence of top management support on green procurement through green training and found that top management is positively associated with green procurement. In a similar vein, Singh et al. (2020) conducts research from the Resource Based View and sees employees as a resource that a leader develops. They looked at the effect of green transformational leadership on environmental performance through the lens of green HRM practices and green innovation. Increased green transformational leadership provides motivation to employees to undertake green innovative activities which in turn lead to better environmental performance.

### Green Innovation

Innovation termed by Schumpeter (Elliott, 1983) refers to the reorganisation of factors of production and can be understood in business practices as changes in products, production process, resources, and managerial organisation form (Chen et al., 2017). Extended from innovation and as a “subset of all innovations” (Kam-Sing Wong, 2012), green innovation refers to innovations that produce products, processes and management structures that minimise resource consumption and lower waste and pollution (Abbas and Sağsan, 2019). This may be done in order to meet policy requirements or to satisfy target customers (Kam-Sing Wong, 2012).

Green innovation has received much attention in recent years and has been studied at many levels, including at the government level, at the industry level and the organisational level. This paper focuses on the organisational level, because at a governmental level, green initiatives and regulations are exterior to an organisation and work as an external pressure on a company (Chen et al., 2018). At an organisational level, green efforts are influenced by the external factors and changes occur within the company. Amongst authors exploring green innovation at an organisational level, green innovation has been studied from different angles. Some authors have looked at the relationship between variables and green innovation in terms of green process and green product innovation (Chen et al., 2006; Kam-Sing Wong, 2012; Lin and Chen, 2017; Xie et al., 2019). Other authors have further distinguished the concept by also exploring green organisational, green marketing, green system and green management innovation (Abdullah et al., 2016; García-Granero et al., 2018). Li et al. (2019) looked at green innovation from a capability perspective by exploring the inputs and outputs of green technological innovation ability. Delgado-Verde et al. (2014) broke innovations into product specific innovations versus innovations throughout whole product lifecycle. Jie et al. (2019) looked at innovations from the angle of cleaner technology, end-of-pipe and resource reduction.

Contrary to research on the effect of green innovation on green environmental performance and total profit indicators, green product success as a performance indicator has received relatively little attention in the literature (Huang and Wu, 2010; Kam-Sing Wong, 2012). Huang and Wu (2010) suggest that new green product development success is important in moving toward further future sustainable activities. If green products are not successful in terms of their profitability, there is no economic motivation pushing companies to continue to undertake innovation activities to develop new green products. In literature, authors define new product success by two dimensions. Firstly, the translation of innovation into a new product, but also how well the new product does on the market (Kam-Sing Wong, 2012).

### Green Product Success

Many authors acknowledge the importance of green innovation in gaining competitive advantage (Kam-Sing Wong, 2012; Chang, 2018; Chu et al., 2019; Takalo et al., 2021), and to achieve greater corporate performance (Chen et al., 2015; Weng et al., 2015; Xie et al., 2019). Much research has consistently concluded that Green Innovation impacts positively on firm performance (Weng et al., 2015; Zhou et al., 2018; Baah et al., 2020; Mohsin et al., 2020; Rehman, 2020; Ma et al., 2021).

Compared with other measures of green success factors, green product success as a performance indicator has received relatively little attention in the literature (Huang and Wu, 2010; Kam-Sing Wong, 2012). Huang and Wu (2010) suggest that new green product development success is important in moving toward further future sustainable activities. If green products are not successful in terms of their profitability, there is no economic motivation pushing companies to continue to undertake innovation activities to develop new green products.

Because products need to be successful in order to continue sustained production in the long term, an important area of research is to look at what makes green products more successful than non-green products. Some authors note cases where, although more and more consumers indicate a preference for products that have lower environmental impact, in reality they often purchase the non-green alternative. Olson (2013) explored the notion of a “value-action gap” in consumers green buying preferences, in other words the disparity between consumers green values and the action of them choosing green products over conventional products. It was found that there are other attributes that are important in buying decisions other than a products greenness, especially if there is a “trade-off” when buying a green product. For example, if buying the green product instead of the conventional product means a reduction in size or performance of the product this represents a trade-off that may mean the consumer will not choose the green product. Products with a unique selling point offer functions not available in competing products. One manifestation of creating products that stand out from competitors is by the creation or improvement of products through innovation.

In literature, authors define new product success by two dimensions. Firstly, the translation of innovation into a new product, but also how well the new product does on the market (Kam-Sing Wong, 2012). Kam-Sing Wong (2012) takes new green product success as a variable in his model and measures it from three dimensions. Firstly, how well the product conforms to the environmental requirements for it to qualify as a green product. Secondly, its financial performance and thirdly, the general perception of the success of the product. The authors research found that there is a significant correlation between green process innovation and new green product success as well as between green product innovation and new green product success.

### Green Brand Image

Green brand image refers to the brand image in terms of its environmental aspects. Kotler’s definition of brand image is “a set of ideas, beliefs and impressions that a customer holds for a certain product or brand” (Kotler and Keller, 2006; Zameer et al., 2020). Therefore, green brand image can be described as the set of ideas, beliefs, or impressions about a firm’s environmental activities.

Green brand image is divided into two parts: functional or tangible; and psychological (Zameer et al., 2020). Green brand image reflects a company’s attitude toward environmental protection, it can also set the company apart from competitors. A strong brand image allows a company to be the first choice when consumers consider buying a product. Customers who have beliefs about the greenness of a product will be more inclined to purchase it over competing products if they perceive that there is no difference between them.

Additionally, Aivazidou et al. (2018) note that a strong green corporate image is important, especially in industries that have high negative externalities and gave the example of firms with high water wastage. Authors noted that it could affect the performance of a firm by noting that firms with a favourable image benefit from greater financial performance.

When researching green brand image, authors have looked at how factors affect green brand image which in turn influences performance indicators. For example, Zameer et al. (2020) explored the mediating role of green image between green production and green competitive advantage. They suggested that green production firstly elevates the green image of the company which then leads to creation of a green competitive advantage. Similarly, Xie et al. (2019) suggested that green activities are only worthwhile for a company when they are sufficiently promoted, when customers have a certain awareness of the greenness of the company and thus feel affinity to a brand. Their research suggested green image has a moderating role on financial performance. Therefore, firms should build up their green image to create a better image in minds of potential customers to improve their performance. As it is an unexplored moderating variable, this research paper will explore the role of brand image in the success of new green products.

## Hypoyhesis Development

**FIGURE 1:**
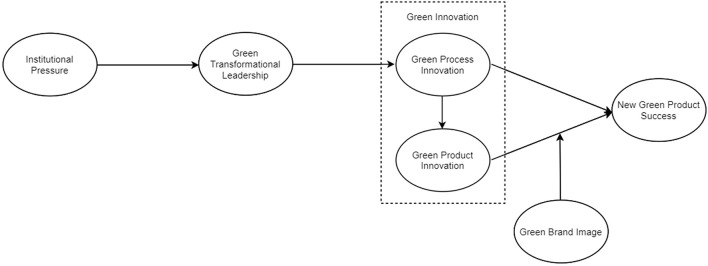
Conceptual model.

A conceptual model was created to help test the relationship between variables, as noted in Figure 1. The model comprises of six variables, discussed below.

### Institutional Pressure and Green Transformational Leadership

Dubey et al. (2015) note that institutional pressure is a driving factor in environmental management practices. Management practices in a company are formed by leadership as they are the principal decision makers and planners in a company. Leaders are the ones who hold ultimate responsibility and not employees for the conduct of the company. Therefore, the response to institutional pressures will be mainly borne by leaders. Failure to comply with government regulations will then mean that company leaders are the ones to face repercussions. The burden of pressure, therefore, falls onto leaders’ shoulders. They weigh up different pressures in creating their vision for the company, consciously or subconsciously, and this can influence their personal inclination toward being a greener company. The greater the external pressure from government regulations, from competitors who are creating greener products that consumers prefer, and from the general societal opinion of the times, the greater a leader will likely be in believing that he or she must adapt and the greater a leader will believe in the importance of being green. It can thus it can be hypothesised that institutional pressure can influence green transformational leadership positively.

As institutional pressures increase, firms seek legitimacy (Qi et al., 2021). This could be because leaders, as the inspiration and providers of the company vision, they are the ones who are primarily the recipients of external pressures and are the main drivers in a firm’s response to pressures. Leaders are the ones who inspire implementations of greater green practices by advocating for greater green activity and green strategy. It is also upon leaders’ shoulders that a burden falls if green requirements or legislation is not met, therefore they are also advocates of meeting external environmental legal requirements. They are the ones who are mostly influenced by external pressures and are the members of a company to take actions to transform the company into a particular direction, such as inspiring greater green behaviours.

Additionally, Chen et al. (2018) found that institutional pressure correlates positively with green innovation. This finding has helped contribute to the growing body of literature in that it shows external pressures can increase levels of green innovation. This paper will test the hypothesis that this happens through the pressure being exerted on leaders, becoming receptive to the pressures. Becoming receptive to these pressures means that leaders will seek to bring about changes in business processes. Thus, we propose the following hypothesis.

*H1: Institutional pressure is positively correlated to green transformational leadership*

### Green Transformational Leadership and Green Process Innovation

A key research area that must be addressed is to look at what affects the level of green innovation companies undertake. This should be addressed because much prior research has focussed on the results on performance of innovation (Kraus et al., 2020). However, there are many unexplored factors that explain what encourages initial green improvements (Chen et al., 2018). Changes to workflows, processes and products fall under the rubric of innovations and changes. Identifying what factors will help bring about changes to business processes can help in encouraging greater green improvements and innovations, which is important in adding to the growing body of green literature, as well as having practical value in encouraging companies to move toward a greener future. Therefore, this section discusses how green transformational leaders can inspire greater innovations.

As leaders are also decision makers in hiring and choose to hire employees whose values align with their own or with company values, if leaders embracing green innovation are highly receptive to embracing green initiatives, then they will seek employees who also possess green values. People are one of the most important resources in a company and the creativity of employees is an important factor in organisational performance (Chang and Hung, 2021). Additionally, employees are directed by leaders who let them give full play to their creativity to meet the aims directed by leaders. Therefore, if leaders are more willing to develop green methods, both leaders and employees will use creativity to develop green improvements, which will lead to the necessary changes to processes and products that can satisfy customers. Leaders are the ones who influence the direction that a company will go in. Employees will be influenced and motivated by the encouragement of leaders, especially on important decisions that may seem to conflict with profit making activity.

Similarly, Chen and Chang (2013) note the important role of leaders in influencing and persuading people to achieve certain goals and encouraging innovation. Leaders act as a “catalyst” that encourage employees to solve problems in new ways. Singh et al. (2020) note that green transformational leaders stimulate product and process innovations through encouraging employees to acquire new knowledge. Previous studies have explored the relationship between institutional pressure and green innovation (Chen et al., 2018), providing more evidence for the hypothesis. As well as inspiring employees’ behaviours and innovation, leaders are also responsible for creating the company values. When hiring new employees, firms ultimately decide to hire those whose values align with the companies. Therefore, leaders with a high level of green consciousness will consciously or subconsciously develop green values within the company. When it comes to hiring employees, they’ll be more likely to employ those with higher levels of green values. In turn they will be more receptive to developing green processes and products and undertaking green activities.

Previous studies have noted that greater institutional pressure leads to greater green innovation (Chen and Chang, 2013) but did not explore the reasons for this, which shows that many authors have supposed this hypothesis but are lacking empirical support for assumptions. This study will therefore explore green transformational leadership as a factor in explaining how institutional pressures can lead to greater innovation. Thus, we propose the following hypothesis.

*H2: Green transformational leadership is positively correlated to green process innovation*

### Green Process Innovation and Green Product Innovation

Green innovation is broken down into process and product innovation as discussed in the literature section. This paper supposes that green transformational leadership affects process innovation as opposed to directly influencing product innovation. For this reason, this paper has distinguished the two concepts in the model. Green process innovations can be seen as those that: use less resources than previous processes before innovations took place; use less resources than competing companies; re-use/re-cycle used materials; use “cleaner” technologies such as renewable energies; and follow and abide by environmental directives. Processes that reduce toxicity and prevent pollution are also green process innovations (Chen et al., 2006; Chen, 2008). Green product innovation is related to changes in products and looks at whether products use less polluting or non-polluting/non-toxic materials, whether the products are made from recycled materials, whether the products are packaged in environmentally friendly packaging and whether the products take into account product end-of-life and disposal.

The reason green product innovations are contingent on green process innovations is because any changes made to a product, such as the change in material used to a recyclable material, means that a change in the processes used to create the product have changed (Xie et al., 2019). In this case, the production line will switch its production process to manufacturing materials that are greener. Additionally, changes in the processes lead to a product that itself is more environmentally friendly. For example, a product that uses less water to produce is now considered a green product. This change did not materialise in the product itself but was a result of changes to production. However, in the consumers eyes, this change has now become a “green” product because its carbon footprint is lower than previous versions or than competitor products. In line with the above reasoning, Kam-Sing Wong (2012) also note that process innovation is a necessary antecedent of product innovation because green process innovations are necessarily involved in green product innovations.

Innovations in processes may result in the creation of new products and help improve the quality of existing products. Xie et al. (2019) posit that green process innovation serves to facilitate green product innovation and not that more focus on green product innovation will shrink the effect of green process innovation, as research prior to that had suggested. The researchers provide empirical analysis to support their view that green process innovation is positively related to green product innovation. This hypothesis has been tested by previous papers, and therefore this paper will test this hypothesis to strengthen evidence and provide more support for the hypothesis. Thus, we propose the following hypothesis.

*H3: Green process innovation is positively correlated to green product innovation*

### Green Process Innovation, Green Product Innovation, and New Green Product Success

As Kam-Sing Wong (2012) note, to succeed in developing new products, one must undertake innovation and have an understanding of market requirements, however, in green innovation one must also have an understanding of environmental ethics. Much research has been conducted on the relationship between innovation and firm performance, all of which show that greater levels of innovation lead to greater firm success. In regard to green innovation, implementing green processes may not be as cost-effective as compared to if non-green methods were implemented. For example, fossil fuels are less expensive and more efficient than switching to wind or solar energy. Therefore, it could be the case that a competing firm could produce a product at a lower cost, but that product is not green. The greener firm could innovate its processes to create better, more cost-effective products, but this may not be as cost-effective as if they had implemented non-green changes. However, companies that make green changes may capture a more green-conscious segment of the market, avoid certain taxes or regulation costs, and gain a green competitive advantage, (Kam-Sing Wong, 2012; Xie et al., 2019) etc. This can mean that, in the end, the payoff of their innovations is greater than those firms that did not undertake green innovations. Li et al. (2019) also noted that higher levels of green technological innovation ability, which refers to the competence of a company to reduce it’s harmful environmental impact, such as energy consumption, lead to greater enterprise competitiveness. This means they are more competitive in customers eyes and customers will be more inclined to purchase the product over competitor products.

Therefore, it is hypothesised that green innovation (and the subcomponents of process and product innovation) have a positive relationship with the success of new green products. In one respect the success of a green product is measured in terms of how successfully the product is, or has become, green. Firms that can reduce their energy use or reduce water waste during the production of new products succeed in creating green products because they have a lower carbon footprint than similar products.

To put it briefly, green product success measures three different aspects. First is the extent to which a new product has been successful in meeting the “green” criteria, for example if it is compliant with environmental directives. Secondly, is the extent to which the new product performs better financially, in terms of revenue and profitability, than competitor products. The final aspect is whether the company views the product as a success or failure. Thus, we propose the following hypothesis.

*H4: green process innovation is positively correlated with new green product success*

*H5: green product innovation is positively correlated with new green product success*

### Moderating Role of Green Brand Image Between Green Product Innovation, and New Green Product Success

Research by Xie et al. (2019) on the moderating role of green image between green product innovation and financial performance supported the idea that green image can work as a moderating variable between green innovation and a success factor of green activities, in their research they looked at financial performance. In this paper we will explore the moderating role on the new products specifically which have been innovated and released on to the market.

In our analysis we research how the success of new green products is heightened by a strong green brand image. If the company does not have a preconceived strong green brand image in the eyes of consumers, new products may not necessarily be successful because consumers make purchasing decisions based on their preconceived ideas about the greenness of the company before they have tried the new products. Consumers may have no idea about all the characteristics of a new product, especially in relation to the greenness of that particular product, and as compared to a similar competing product. From looking simply at the product, packaging and brief information about the product in question, it may not be clear how the product competes with others in terms of its greenness. It may not even be as green as a competitor product. Consumers therefore use their pre-existing impressions of the companies’ values to help them in deciding if the company aligns with their own values and whether it is worth buying from over a competitor. They take cues from their impression of the company to assess whether the product they are considering buying is as green as the product information and packaging supposes when compared to competitor products. When comparing two products, its greenness will not be the only factor in the purchasing decision, but for products who have the same functions, a customer will use their impression of a company as an aid. Companies who are favourable in the eyes of a consumer in terms of the company’s greenness will be seen to have a greener product, by brand association, even if for that particular product a competitor product was greener. Therefore, green brand image is an important factor in mediating green product innovations and how successful the resultant new products perform on the market.

Although green innovations may lead to an improved brand image and in turn lead to higher success of products, this paper will not research green brand image as a mediating variable but research how the green brand image moderates the success of new green products. We hypothesise that if green brand image is high, then customers will more likely view the company’s new products as green which will mean that when they have a greener impression of the company, they will be more likely to buy the new green products. green brand image is a variable that has been built up by various marketing activities of the company and influences purchases of old products also. New green product success is not only affected by the innovation of new green products, hence we explore the variable as a moderating variable. Thus, we propose the following hypothesis.

*H6: Green brand image moderates the relationship between green process innovation and green product success such that the positive relationship is stronger when green brand image is high-level rather than low-level*

## Methodology

### Sampling and Data Collection

To test hypotheses, this paper used a questionnaire method to collect data. This method allows for a large sample size that is required to draw meaningful conclusions. The respondents were drawn from Chinese mainland companies. This is because the researchers were located in China and the questionnaire language was in Chinese. The Chinese government is increasing its efforts in green technologies and encouraging and regulating companies to increase green activity, which, as previously stated, tend to filter down into leaders visions and ultimately management practices. A questionnaire was used as a means of gathering data over other methods because it allowed for a greater number of respondents, because a greater number of potential respondents could be accessed online and via mail. Due to the ease of a short questionnaire, the number of respondents was greater than methods such as an interview. Since the questionnaire measured variables such as firm performance, real life behavioural simulation activities were not appropriate. Lastly, although information on new green product success could have been collected from company reports, some variable measurements would not have been as readily available, such as measurement of green transformational leadership. Questionnaire was therefore deemed the most appropriate method of data collection. There were no sampling methods used for this test because all respondents targetted were deemed appropriate to take part in the survey.

This paper uses structural equation modelling to test our hypothesis. Using a questionnaire, this paper collected data to measure the variables. To measure variables, they were first operationalised using scales borrowed from prior literature. The questions from the scales were then collated into a questionnaire with other questions such as company size, location and industry.

The questionnaire was issued to companies in first, second and third tier cities in mainland China over a 2-month period in the spring of 2020. Relevant managers enrolled in the Qingdao University MBA programme were identified and targetted to participate in the study via a link to an online questionnaire platform named *Wenjuanxin*, which ensured that no questionnaires could be sent incomplete and allowed for easier collection of data. Additionally, questionnaire invitations were sent out via email to 300 managers in cities whose companies were located in first, second and third tier cities in Mainland China. The total number of responses was 243.

### Measurement of Variables

To measure variables, scales were adopted from previous literature. The questions were compiled and put on a 7-point Likert scale, ranging from 1 for strongly disagree to 7 for strongly agree. A pilot test was then conducted to ensure the validity and reliability of the questions to ensure that they were appropriate for use in the final questionnaire and that they did not need changing. The original questionnaire is available in the Supplementary Material.

### Institutional Pressure

The scale for institutional pressure was taken from Dubey et al. (2015) and was created with the following items: (1) Is regional pollution control board pressuring the firm to adopt green practices (2) Do Government regulations provide clear guidelines in controlling pollution levels (3) Does the pollution control board strictly monitor pollution level of the firm on a regular basis (4) Do green practices decrease incidences of penalty fees charged by the pollution control board (5) Are maximum sales of the company export oriented (6) Are foreign customers more sensitive toward green practices.

### Green Transformational Leadership

The scale for institutional pressure was taken from Dubey et al. (2015) and was created with the following items: (1) Does your company have a well-defined environmental policy (2) Is every employee aware about the firms environmental policy (3) Does top management support environmental programmes (4) Top management has approved special funds for investment in cleaner technologies (5) Do senior managers motivate and support new ideas received from junior executives (6) Are employees recognised for innovative ideas and awarded on a periodic basis.

### Green Process Innovation

The measurement of green process innovation is measured using the scale taken from Kam-Sing Wong (2012). In total there are six questions, as follows: (1) Our production processes consume less resource (e.g., water, electricity, etc.) than those of our competitors (2) Our production processes recycle, reuse and remanufacture materials or parts (3) Our production processes use cleaner or renewable technology to make savings (such as energy, water and waste) (4) We redesign our production and operation processes to improve environmental efficiency (5) We redesign and improve our products or services to meet new environmental criteria or directives (such as WEEE directive, RoHS directive, etc.).

### Green Product Innovation

The measurement of green process innovation is measured using the scale taken from Kam-Sing Wong (2012). In total there are four questions as follows: (1) Our new products use less or non-polluting/toxic materials (2) Our new products use environmentally friendly packing (3) When designing new product, we take recycling and disposal at end of life into account (4) Our new products use recycled materials (5) Our new products use materials that have been recycled.

### New Green Product Success

The measurement of New Green Product Success is also measured using the scale taken from Kam-Sing Wong (2012). There is a total of five questions related to the success of the product as a “green” product, the success on the market and the opinion of leaders concerning product’s success. The items are as follows: (1) Our green new products are in compliance with environmental directives (2) Our green new products meet the environmental requirements set by stakeholders (3) Our green new products bring in more revenue than competing products (4) Our green new products are more profitable than the competing products (5) Our green new products are successful.

### Green Brand Image

The measurement of green brand image is measured using the scale taken from Zameer et al. (2020). The items are as follows (1) The brand is regarded as a benchmark of environmental commitments (2) the brand is professional about environmental reputation (3) The brand is successful about environmental performance (4) The brand is well established about environmental concern (5) The brand is trustworthy about environmental promises.

### Pilot Test

A pilot test was carried out by identifying and approaching members of the Qingdao University MBA programme who worked in companies located in Shandong, China. 102 responses were received and analysed. The scales were tested for reliability and validity to ensure the reliability of the scales. Items in all four variables were tested for reliability and the Cronbach Alpha are as follows for institutional pressure, Green transformational leadership, Green Process Innovation, green product innovation, Green Image and Green Product Success respectively: 0.877, 0.832, 0.776, 0.791, 0.712. All of these are acceptable reliability scores. This meant that the scales borrowed could be used in the final questionnaire.

## Empirical Results

As discussed in the previous section, the final questionnaire was issued to company managers in mainland China between May to June 2020. The number of responses received was 243. Descriptive results were first analysed to draw a general picture of the data. Scales were then tested again for reliability and validity and then a confirmatory factor analysis was conducted. Tests of model fit were conducted to ensure that the results drawn about hypotheses were meaningful, that variables measured what authors wanted them to and that multicollinearity in variables did not pose a problem in drawing conclusions from the data.

### Descriptive Statistics

The mean size of the company was 2.71. 1 was a company with between 1 and 25 employees, 2 was a company with between 26 and 50 employees, 3 was a company with between 51 and 100 employees (1 = 1–25, 2 = 26–50, 3 = 51–100). Therefore, the average size of the company was between 26 and 50 employees. As for the industry, the majority of respondents worked in the IT/software/internet industry, with the number of respondents in the industry being 43, representing 17.7% of the total. This was followed by 37 respondents who worked in the manufacturing industry, representing 15.2% of the total respondents. Respondents working in import/exports represented 8.6% of the respondents, with the number of respondents being 21, and respondents in the telecoms and networks represented 7.8% of the respondent total, with 19 respondents in that industry. This was the same respondent count as those working in the furniture craft industry, which also represented 7.8%.

### Reliability and Validity

A reliability test measures how closely related a set of items in a scale are and can be likened to a dartboard onto which arrows have been projected, how close the arrows are to each other represents the reliability of the items, each item is measuring the same thing. Validity measures how well the measurement of a concept corresponds to what is being measured in the real world. Does it achieve the measurement of the concept it claims to measure? Similarly, this can be likened to a dartboard, this time measuring whether the arrows are landing on the dartboard or not. If all the dartboard arrows are hitting the same spot but the arrows fall next to, and not on, the dartboard then the questions are reliably measuring the same thing, but the concept measured does not measure the variable that the study wishes to measure, therefore does not achieve validity.

As we did in our pilot test, we carried out validity and reliability tests again on the scales using IBM SPSS 20. The Cronbach Alpha are as follows for institutional pressure, green transformational leadership, green process innovation, green product innovation, green brand image and green product success respectively: 0.747, 0.904, 0.945, 0.956, 0.861, 0.735, all of which are acceptable scores for testing our model. Scales for all variables were tested achieving the following KMO scores. institutional pressure (0.827), green transformational leadership (0.902), green process innovation (0.899), green product innovation (0.905), green brand image (0.803) and green product success (0.758). All scores are acceptable for testing our model.

### Confirmatory Factor Analysis

Confirmatory factor analysis allows us to assess whether our questionnaire is acceptable to measure our constructs and was performed using SMARTPLS3. We tested the model for fit and received an SRMR score of 0.0769, a score <0.08 is considered an acceptable fit. We then used Confirmatory Factor Analysis to test loadings as shown in Table 1. T statistic and *P* values show the variables in the model to be significant. The results indicate that our constructs are an accurate measure of variables and we can proceed with further analysis. Table 2 contains the results of the confirmatory factor analysis conducted in Mplus 7.4, which shows the fitting indexes of the six-factor model constructed in this paper are better than those of other factor models, which shows that the model constructed in this paper has good construction validity and good discrimination validity among variables.

**TABLE 1:** Confirmatory factor analysis.

Construct	Items	β	Sample mean (M)	Standard deviation (STDEV)	T statistics (| O/STDEV|)	*P* values
Green process innovation	GPROCI1	0.88	0.88	0.04	22.03	0
	GPROCI2	0.88	0.88	0.04	24.26	0
	GPROCI3	0.89	0.89	0.03	29.38	0
	GPROCI4	0.9	0.9	0.03	25.94	0
	GPROCI5	0.85	0.85	0.04	21.73	0
Green product innovation	GPRODI1	0.94	0.94	0.04	21.77	0
	GPRODI2	0.92	0.92	0.02	38.76	0
	GPRODI3	0.82	0.82	0.05	17.57	0
	GPRODI4	0.9	0.9	0.03	31.99	0
	GPRODI5	0.92	0.92	0.03	36.28	0
Green product success	GPS1	0.72	0.72	0.07	10.94	0
	GPS2	0.7	0.7	0.06	12.09	0
	GPS3	0.41	0.41	0.08	5.16	0
	GPS4	0.63	0.63	0.06	9.79	0
	GPS5	0.42	0.42	0.08	5.37	0
Green transformational leadership	GTL1	0.64	0.64	0.15	4.26	0
	GTL2	0.51	0.52	0.18	2.9	0
	GTL3	0.84	0.82	0.15	5.62	0
	GTL4	0.91	0.88	0.12	7.61	0
	GTL5	0.95	0.91	0.11	8.47	0
	GTL6	0.74	0.73	0.12	6.01	0
Institutional pressure	IP1	0.79	0.77	0.07	12.19	0
	IP2	0.52	0.52	0.09	5.96	0
	IP3	0.37	0.36	0.09	3.92	0
	IP4	0.45	0.45	0.09	5.03	0
	IP5	0.41	0.41	0.1	4.2	0
	IP6	0.47	0.46	0.09	5.47	0

**TABLE 2:** Analysis results of confirmatory factor analysis for the measures of the variables studied.

	χ ^2^	*df*	RMSEA	CFI	TLI	SRMR
Six-factor model	848.06	449	0.061	0.923	0.915	0.085
Five-factor model^a^	1162.57	454	0.080	0.863	0.850	0.123
Four-factor model^b^	1904.39	458	0.114	0.721	0.697	0.132
Three-factor model^c^	2619.48	461	0.139	0.583	0.551	0.143
Two-factor model^d^	3043.41	463	0.152	0.502	0.466	0.143
One-factor model^e^	3161.19	464	0.155	0.479	0.443	0.141

**n* = 243.*

*^*a*^Institution pressure and green transformational leadership merged as a potential factor.*

*^*b*^Institution pressure, green transformational leadership and green brand image merged as a potential factor.*

*^*c*^Institution pressure, green transformational leadership, green process innovation and green product innovation merged as a potential factor.*

*^*d*^Institution pressure, green transformational leadership, green process innovation, green product innovation and green brand image merged as a potential factor.*

*^*e*^All measurements merged as a potential factor.*

### Model Fit

To test for Common Method Bias, we assessed the Variance Inflation Factor (VIF) values. The Variance Inflation Factor tests for multicollinearity, which occurs when the X variables are related, skewing the results because it is not possible to hold one variable constant in measuring if it is affected itself by other X variables. A Variance Inflation Factor (VIF) test is run, in which for each variable it is regressed onto the other variables in the model. This essentially looks at how much X1 is explained by the other X variables. The VIF is calculated by taking the r^2^ from each regression. For this model, a VIF factor below 3 indicates that multicollinearity does not pose a problem in the model. Green brand image to green product success = 2.68862, green brand image moderator to green product success = 2.717653, green process innovation to green product success = 2.401532, green product innovation to green product success = 2.155512. Values of less than 3.3 indicate that multicollinearity does not represent a problem in our model, therefore we can accept our model.

We then used the Heterotrait-Monotrait ratio to assess discriminant validity which analyses the relationship between latent variables. Values less than 1 are acceptable. As shown in Table 3, all values are less than one, meaning our model is acceptable.

**TABLE 3:** Heterotrait-Monotrait ratio values, assessing discriminant validity.

	Green brand image	Green brand image moderator	Green process innovation	Green product innovation	Green product success	Green transformational leadership
Green brand image						
Green brand image moderator	0.583929					
Green process innovation	0.568942	0.059485				
Green product innovation	0.423778	0.199466	0.677122			
Green product success	0.521946	0.229615	0.641922	0.658636		
Green transformational leadership	0.097131	0.172363	0.179389	0.132877	0.176316	
Institutional pressure	0.509764	0.243258	0.408544	0.419572	0.501402	0.262272

### Correlation Analysis

We carried out a correlation analysis in SMARTPLS3. The Correlation Coefficient measures the degree of linear relationship between two variables. We used the Pearson Correlation and results show that all the variables in our model show a correlation at the 0.05 significant level or lower, expect for green transformational leadership to green product innovation and green transformational leadership to green brand image. As outlined in Table 4, results show that all other variables are positively correlated, this means that an increase in one variable is explained by an increase in another, as we predicted in our hypotheses.

**TABLE 4:** Pearson correlations.

Correlations						
	IP	GTL	GPROCI	GPRODI	GBI	GPS
IP						
GTL	0.193**					
GPROCI	0.336**	0.169**				
GPRODI	0.344**	0.122	0.643**			
GBI	0.391**	0.078	0.512**	0.384**		
GPS	0.356**	0.147*	0.535**	0.551**	0.422**	

*Correlation is significant at the 0.05 level (2-tailed).

**Correlation is significant at the 0.01 level (2-tailed).

### Hypothesis Testing Using Structural Equation Modelling

We tested the model using structural equation modelling using SMARTPLS3 and SPSS2.0. The results can be seen in Table 5. The path coefficients are reported under β, the T-statistic and P-value are also presented in the table for each of our hypotheses.

**TABLE 5:** Hypothesis test results.

H		β	T statistic	*P* values	Supported
H1	Institution pressure to green transformational leadership	0.299	3.796	0.000	Supported
H2	Green transformational leadership to green process innovation	0.188	2.691	0.007	Supported
H3	Green process innovation to green product innovation	0.678	16.027	0.000	Supported
H4	Green process innovation to green product success	–0.007	0.037	0.971	Reject
H5	Green product innovation to green product success	0.493	2.691	0.007	Supported
H6	Green brand image moderator	0.580	11.063	0.000	Supported

Institutional pressure is positively correlated with green transformational leadership, showing support for hypothesis one (H1) that institutional pressure is positively correlated with green transformational leadership, this could be due to leaders taking certain actions as a result of seeking legitimacy from institutions in their field, which exert pressure on a company’s actions (Chen et al., 2018). For example, leaders may be conscious of government green regulations, and will anticipate how conforming or not confirming could affect the company, and then will encourage the development of business processes and product development accordingly, so as to conform to the regulations. Some leaders may also see that competitors are conforming and going further in their green innovations and so want to keep up with and outperform competitors. Therefore, they may also believe that setting an example of green leadership is important in order to stay competitive in the market.

Green transformational leadership then has a positive correlation with green process innovation, providing support for hypothesis two (H2) which shows that green transformational leadership can possibly influence green process innovation as leaders are instrumental in encouraging employee’s green innovation spirit. Leaders make the decisions of the direction of the company, therefore, if they come to believe that green innovation is important for the company to survive, or they have been influenced by societal pressure to believe that green is the general trend toward which companies should advance, then they will advocate for such a greener direction within the company accordingly, whilst still maintaining the necessity to make a profit. Leaders with a strong green mindset will seek to improve processes in a green way. Leaders also set the example for employees. If they believe in green ideals and a green future, they may advocate this through policies and trainings. This can influence how important employees take green development within the company. Secondly, leaders who are more green-focussed will most likely find employees who have similar values to the company.

Green process innovation is positively correlated with green product innovation, providing more support for hypothesis three (H3). This hypothesis was also supported by Xie et al. (2019). This indicates that greater green process innovation enhances green product innovation because changes made to processes can help to produce new products that could not be created unless there where changes in the processes used to make new products. When new green products are designed, changes must necessarily be made to the processes in order to create a new green product, such as designing a product that uses a different material may mean that less of the material needs to be used and the factory will adjust its manufacturing method to switch to this new material and manufacturing process. Additionally, when an innovation in a production process occurs, it can mean that the product that results from the production process will be one that, for example, uses less electricity to produce or produces less waste during production. Therefore, the product has a lower carbon footprint that previous product designs, and if it is released as a new version, can be considered a new green product.

In our model, green process innovation is not shown to have a positive correlation with green product success (H4) and the result is not significant. Therefore, we rejected this hypothesis. The reason for no positive correlation being found may be that some innovation in processes may not affect the functions of the product directly. For example, some process innovations may switch to using greener forms of energy to power machines and not fossil fuels. From its exterior appearance, this would not change the product directly. Although the product itself can now be considered a new green product from the perspective of innovation. In consumers eyes the changes may not be evident, and thus the performance of the product won’t necessarily be successful. It is innovations in the methods used to create the same products, and thus in consumers eyes they will have no more incentive to purchase the product due to no perceived improvement in functions. This contrasts with the findings by Kam-Sing Wong (2012) and therefore may warrant further research to determine under which circumstances, or which type of changes in process, innovation leads to product success.

There is a strong correlation between green product innovation and new green product success, which supports hypothesis five (H5) and indicates that green innovations in products enhance the success of these products when released onto the market. Products that are highly innovative are perceived by consumers as superior to alternatives. If the products are also environmentally friendly then customers, who also live in a society which is increasingly conscious of human degradation of the planet, will also take the green component of the product into account in his or her purchase decision. Products with better functions will be more successful than competitors because consumers value them over competitors. Products that have successfully changed from a non-green or highly polluting product to a greener and less polluting product will also be seen as successful in terms of the state of greenness of the product itself. Therefore, green product innovations lead to successful green new products.

Lastly, we tested our moderating variable green brand image between green product innovation and new green product success. This was tested using SPSS 22.0 and Mplus 7.4. The moderating variable shows that a 58% change in green product success is due to the moderator variable (β = 0.580). Green brand image moderates the relationship between green process innovation and green product success such that the positive relationship is stronger when green brand image are high-level rather than low-level. We can therefore accept the hypothesis. Green brand image moderates the role between green product innovation and new green product success. Green brand image moderates the relationship between green process innovation and green product success, such that the positive relationship is stronger when green brand image is high-level rather than low-level. This indicates that when green brand image is higher, green product success will likely be higher too, when green brand image is lower, there is not significant effect on green product success. This is because when new products come onto the market, if they are green but customers are not aware of their green status, they will not be more inclined to choose such products over competitors, as they do not perceive them as superior to competitor products. Products that are associated with greenness are more likely to be chosen over competitor products. For a company with a strong green brand image, customers are more likely to associate a new product with possessing green qualities as compared to a company that doesn’t have a strong green brand image. In this case consumers will less likely perceive the competitor green product as green, despite the fact it may be greener. Because consumers impression of a brand plays a large role in a purchase decision, this hypothesis has shown that green brand image is important in the success of a new green product.

## Discussion and Conclusion

This study attempted to answer how institutional pressures can affect the success of new products on the market that are considered to be green. It looked through the lens of green innovation and explored how a high green brand image can affect the success of green products. A total of 243 managers were sampled and the results of the analysis show that institutional pressure can positively affect the extent to which leaders inspire green behaviours in a company. The study shows that higher encouragement from green leaders suggests that the level of green innovation undertaken will be greater. Greater levels of green innovation were shown to lead to a greater success of new green products on the market, because products developed have better features than past versions or competitors and are more appealing to customers. The study also showed that the brand image of a company affects the success of the new products. The greener a company is seen in general, the greater the extent new customers will be receptive to buying the new green product.

### Theoretical Contributions

This paper has made several theoretical contributions as outlined below. Firstly, it has explored how institutional pressure influences green transformational leadership and how it in turn affects green innovation. It was found that institutional pressure has a positive correlation with transformational leadership, which in turn influences green innovation positively. This is in line with previous research where Chen et al. (2018) looked at institutional pressure and green innovation and Singh et al. (2020) looked at the relationship between green transformational leadership and green innovation.

This paper also explored the moderating role of green brand image between green product innovation and green product success and found that green brand image moderates green product innovation and new green product success. That is to say that when a company’s green brand image is high, then new green products are more likely to be successful that new products in companies whose green brand image is low.

### Practical Implications

From the results we have further support for all our hypotheses bar one (green process innovation to green product success). From a practical management angle, we can make the following conclusions, outlined below. Companies with leaders that have a green inclination will help stimulate green innovations in processes that will help the company to create green products that are more successful in terms of market and environmental performance. The implications of the results will help leaders when deciding which kind of employees they want to employ. Leaders that are more inclined toward green practices are a positive example for employees that may encourage green behaviours, green creativity and green innovation (Singh et al., 2020). If companies want to launch successful green products, they should look for leaders who have an environmentally conscious mindset.

Secondly, this research has highlighted the importance of improving the brand image of a company to reflect its greenness. If companies launch new green products but customers do not believe the company is green, or is environmentally conscious, then when faced with competitor products, customers may not necessarily choose the product because they are not aware of the green qualities. Therefore, when a company innovates new green products and releases them onto the market, they should also rely on building up the brand image in general and not only creating a green product to improve the success of the product. When new green products are launched onto the market, if the firm has a strong green brand image then new products are likely to be more successful than products in companies that do not have a strong brand image. Therefore, leaders should also encourage marketing managers to create marketing strategies and campaigns that help build up a green image in customers minds. With an impression of the company as a whole as a green company, they will be more likely to buy a new green product released on the market by the company.

### Limitations of Research and Future Research

The research was conducted on companies in mainland China, which has a culture different to other countries which may be a limitation of the research. Also, green awareness in consumers’ and leaders’ minds may be different in Western and Chinese societies. Future research could include companies outside of mainland China. Therefore, a future study could be replicated internationally, or in a different country to minimise the impact of cultural difference on responses. Secondly, green innovation was explored by looking at green process and product innovation and did not explore management innovation that has been operationalised in previous research, future research could incorporate a greater number of variables to explore green innovation. Lastly, there was no significant correlation found between green process innovation and green product success, which is contrary to previous research. Therefore, more research could be conducted to explore under what circumstances green process innovations lead to success of new green products if there are no obvious changes to green product functionality or aesthetic.

## Data Availability Statement

The raw data supporting the conclusions of this article will be made available by the authors, without undue reservation.

## Author Contributions

JZ adjusted the framework, analysed the results, and proofread the accuracy of the manuscript. LS proposed the initial research framework, analysed the results, wrote, and proofread the manuscript. AS analysed the results.

## Conflict of Interest

The authors declare that the research was conducted in the absence of any commercial or financial relationships that could be construed as a potential conflict of interest. The reviewer MU declared a shared affiliation with the authors to the handling editor at time of review.

## Publisher’s Note

All claims expressed in this article are solely those of the authors and do not necessarily represent those of their affiliated organizations, or those of the publisher, the editors and the reviewers. Any product that may be evaluated in this article, or claim that may be made by its manufacturer, is not guaranteed or endorsed by the publisher.
