# Supplementary material for: Institutional Pressure and Green Product Success: The Role of Green Transformational Leadership, Green Innovation, and Green Brand Image
Source: Front Psychol. 2021 Oct 4;12:704855. doi: 10.3389/fpsyg.2021.704855 (PMC8520918; doi:10.3389/fpsyg.2021.704855)
Supplement: Supplementary file 1 [file Data_Sheet_1.docx]

Appendices:

Appendix 1: Questionnaire

n.b. translated from Chinese into English

| **Survey on Green Transformational Leadership and Green Product Success** | |
| --- | --- |
|  |  |

|  |
| --- |
| **1.** Choose your location: |
|  |
| _________________________________ |
|  |
| **2.** Your current industry: |
| ○ IT/software/e-commerce/internet operations    ○ Fast moving consumer goods (food, drinks, make up)    ○ Distribution/retail    ○  Clothing/textiles/leather    ○  Furniture/handicrafts    ○  Education/training/school    ○  Household appliances    ○  Telecoms/electronic operations/network equipment    ○  Manufacturing    ○  Automobile & parts    ○  Hospitality/entertainment/travel    ○  Office supplies & equipment    ○  Accounting    ○  Law    ○  Banking/insurance/securities/investment banking/risk runds    ○  Electronic technology/semiconductors    ○  Industrial automation    ○  Import/export    ○  Heavy machinery    ○  Pharmaceutical / bioengineering / medical equipment / devices    ○  Medical / nursing / health care / hygiene    ○ Advertising / public relations / media / art     ○ Publishing / printing / packaging     ○ Real estate development / construction engineering / decoration / design     ○ Property management / business center     ○ Intermediary / consulting / headhunting / certification     ○ Transportation / transportation / logistics     ○ Aerospace / aviation / energy / chemical industry     ○ Agriculture / fisheries / forestry     ○ Other |
|  |
| **3.** The number of employees in your company: |
| ○ 1-25    ○ 26-50    ○ 51-100    ○ 101-200    ○ 201-300    ○ 300-500    ○ 500+ |
|  |
| **4.**  According to the level of institutional pressures faced by your company, please score the below questions with 1 (strongly disagree) to 7 (strongly agree)  Strongly Agree 1------- 2------- 3------- 4 ------- 5 ------- 6 -------7 Strongly Disagree |
| \|  \| 1 \| 2 \| 3 \| 4 \| 5 \| 6 \| 7 \| \| --- \| --- \| --- \| --- \| --- \| --- \| --- \| --- \| \| Is regional pollution control board pressuring the firm to adopt green practices \| ○ \| ○ \| ○ \| ○ \| ○ \| ○ \| ○ \| \| Do Government regulations provide clear guidelines in controlling pollution levels \| ○ \| ○ \| ○ \| ○ \| ○ \| ○ \| ○ \| \| Does the pollution control board strictly monitor pollution level of the firm on a regular basis \| ○ \| ○ \| ○ \| ○ \| ○ \| ○ \| ○ \| \| Do green practices decrease incidences of penalty fees charged by the pollution control board \| ○ \| ○ \| ○ \| ○ \| ○ \| ○ \| ○ \| \| Are maximum sales of the company export oriented \| ○ \| ○ \| ○ \| ○ \| ○ \| ○ \| ○ \| \| Are foreign customers more sensitive towards green practices \| ○ \| ○ \| ○ \| ○ \| ○ \| ○ \| ○ \| |
|  |
| **5.**  According to the level of green transformational leadership in your company, please score the below questions with 1 (strongly disagree) to 7 (strongly agree)  Strongly Agree 1------- 2------- 3------- 4 ------- 5 ------- 6 -------7 Strongly Disagree |
|  |
| \|  \| 1 \| 2 \| 3 \| 4 \| 5 \| 6 \| 7 \| \| --- \| --- \| --- \| --- \| --- \| --- \| --- \| --- \| \| Does your company have a well-defined environmental policy \| ○ \| ○ \| ○ \| ○ \| ○ \| ○ \| ○ \| \| Does top management support environmental programs \| ○ \| ○ \| ○ \| ○ \| ○ \| ○ \| ○ \| \| Is every employee aware about the firms environmental policy \| ○ \| ○ \| ○ \| ○ \| ○ \| ○ \| ○ \| \| Top management has approved special funds for investment in cleaner technologies \| ○ \| ○ \| ○ \| ○ \| ○ \| ○ \| ○ \| \| Do senior managers motivate and support new ideas received from junior executives \| ○ \| ○ \| ○ \| ○ \| ○ \| ○ \| ○ \| \| Are employees recognised for innovative ideas and awarded on a periodic basis \| ○ \| ○ \| ○ \| ○ \| ○ \| ○ \| ○ \| |
|  |
| **6.**  According to the level of green process innovation in your company, please score the below questions with 1 (strongly disagree) to 7 (strongly agree)  Strongly Agree 1------- 2------- 3------- 4 ------- 5 ------- 6 -------7 Strongly Disagree |
| \|  \| 1 \| 2 \| 3 \| 4 \| 5 \| 6 \| 7 \| \| --- \| --- \| --- \| --- \| --- \| --- \| --- \| --- \| \| Our production processes consume less resource (e.g. water, electricity, etc.) than those of our competitors \| ○ \| ○ \| ○ \| ○ \| ○ \| ○ \| ○ \| \| Our production processes recycle, reuse and remanufacture materials or parts \| ○ \| ○ \| ○ \| ○ \| ○ \| ○ \| ○ \| \| Our production processes use cleaner or renewable technology to make savings (such as energy, water and waste) \| ○ \| ○ \| ○ \| ○ \| ○ \| ○ \| ○ \| \| We redesign our production and operation processes to improve environmental efficiency \| ○ \| ○ \| ○ \| ○ \| ○ \| ○ \| ○ \| \| We redesign and improve our products or services to meet new environmental criteria or directives (such as WEEE directive, RoHS directive, etc.). \| ○ \| ○ \| ○ \| ○ \| ○ \| ○ \| ○ \| |
|  |
| **7.**  According to the level of green product innovation in your company, please score the below questions with 1 (strongly disagree) to 7 (strongly agree)  Strongly Agree 1------- 2------- 3------- 4 ------- 5 ------- 6 -------7 Strongly Disagree |
| \|  \| 1 \| 2 \| 3 \| 4 \| 5 \| 6 \| 7 \| \| --- \| --- \| --- \| --- \| --- \| --- \| --- \| --- \| \| Our new products use less or non-polluting/toxic materials \| ○ \| ○ \| ○ \| ○ \| ○ \| ○ \| ○ \| \| Our new products use environmentally friendly packing \| ○ \| ○ \| ○ \| ○ \| ○ \| ○ \| ○ \| \| When designing new product, we take recycling and disposal at end of life into account \| ○ \| ○ \| ○ \| ○ \| ○ \| ○ \| ○ \| \| Our new products use recycled materials \| ○ \| ○ \| ○ \| ○ \| ○ \| ○ \| ○ \| \| Our new products use materials that have been recycled \| ○ \| ○ \| ○ \| ○ \| ○ \| ○ \| ○ \| |
| **6.**  According to the green brand image of your company, please score the below questions with 1 (strongly disagree) to 7 (strongly agree)  Strongly Agree 1------- 2------- 3------- 4 ------- 5 ------- 6 -------7 Strongly Disagree |
| \|  \| 1 \| 2 \| 3 \| 4 \| 5 \| 6 \| 7 \| \| --- \| --- \| --- \| --- \| --- \| --- \| --- \| --- \| \| The brand is regarded as a benchmark of environmental commitments \| ○ \| ○ \| ○ \| ○ \| ○ \| ○ \| ○ \| \| The brand is professional about environmental reputation \| ○ \| ○ \| ○ \| ○ \| ○ \| ○ \| ○ \| \| The brand is successful about environmental performance \| ○ \| ○ \| ○ \| ○ \| ○ \| ○ \| ○ \| \| The brand is well established about environmental concern \| ○ \| ○ \| ○ \| ○ \| ○ \| ○ \| ○ \| \| The brand is trustworthy about environmental promises \| ○ \| ○ \| ○ \| ○ \| ○ \| ○ \| ○ \| |
|  |
| **6.**  According to the success of new green products in your company, please score the below questions with 1 (strongly disagree) to 7 (strongly agree)  Strongly Agree 1------- 2------- 3------- 4 ------- 5 ------- 6 -------7 Strongly Disagree |
| \|  \| 1 \| 2 \| 3 \| 4 \| 5 \| 6 \| 7 \| \| --- \| --- \| --- \| --- \| --- \| --- \| --- \| --- \| \| Our green new products are in compliance with environmental directives \| ○ \| ○ \| ○ \| ○ \| ○ \| ○ \| ○ \| \| Our green new products meet the environmental requirements set by stakeholders \| ○ \| ○ \| ○ \| ○ \| ○ \| ○ \| ○ \| \| Our green new products bring in more revenue than competing products \| ○ \| ○ \| ○ \| ○ \| ○ \| ○ \| ○ \| \| Our green new products are more profitable than the competing products \| ○ \| ○ \| ○ \| ○ \| ○ \| ○ \| ○ \| \| Our green new products are successful \| ○ \| ○ \| ○ \| ○ \| ○ \| ○ \| ○ \| |
|  |
| **10.** Does your company receive government subsidies |
| ○ Yes, my company receive subsidies    ○ No, my company doesn’t receive subsidies |
